# Impact of Clot Shape on Successful M1 Endovascular Reperfusion

**DOI:** 10.3389/fneur.2021.642877

**Published:** 2021-02-01

**Authors:** Adrien Guenego, Robert Fahed, Eric S. Sussman, Matthew Leipzig, Gregory W. Albers, Blake W. Martin, David G. Marcellus, Gabriella Kuraitis, Michael P. Marks, Maarten G. Lansberg, Max Wintermark, Jeremy J. Heit

**Affiliations:** ^1^Interventional and Diagnostic Neuroradiology, Stanford Medical Center, Stanford, CA, United States; ^2^Division of Neurology, Department of Medicine, Ottawa Hospital Research Institute, The Ottawa Hospital, University of Ottawa, Ottawa, ON, Canada; ^3^Stanford Stroke Center, Stanford University School of Medicine, Stanford, CA, United States

**Keywords:** stroke, thrombectomy, endovascular recanalization, magnetic resonance imaging, clot

## Abstract

**Objectives:** The susceptibility-vessel-sign (SVS) allows thrombus visualization, length estimation and composition, and it may impact reperfusion during mechanical thrombectomy (MT). SVS can also describe thrombus shape in the occluded artery: in the straight M1-segment (S-shaped), or in an angulated/traversing a bifurcation segment (A-shaped). We determined whether SVS clot shape influenced reperfusion and outcomes after MT for proximal middle-cerebral-artery (M1) occlusions.

**Methods:** Between May 2015 and March 2018, consecutive patients who underwent MT at one comprehensive stroke center and who had a baseline MRI with a T2^*^ sequence were included. Clinical, procedural and radiographic data, including clot shape on SVS [angulated/bifurcation (A-SVS) vs. straight (S-SVS)] and length were assessed. Primary outcome was successful reperfusion (TICI 2b-3). Secondary outcome were MT complication rates, MT reperfusion time, and clinical outcome at 90-days. Predictors of outcome were assessed with univariate and multivariate analyses.

**Results:** A total of 62 patients were included. 56% (35/62) had an A-SVS. Clots were significantly longer in the A-SVS group (19 mm vs. 8 mm *p* = 0.0002). Groups were otherwise well-matched with regard to baseline characteristics. There was a significantly lower rate of successful reperfusion in the A-SVS cohort (83%) compared to the S-SVS cohort (96%) in multivariable analysis [OR 0.04 (95% CI, 0.002–0.58), *p* = 0.02]. There was no significant difference in long term clinical outcome between groups.

**Conclusion:** Clot shape as determined on T2^*^ imaging, in patients presenting with M1 occlusion appears to be a predictor of successful reperfusion after MT. Angulated and bifurcating clots are associated with poorer rates of successful reperfusion.

## Statistical Analysis

Adrien Guenego, MD and Matthew Leipzig, BS conducted all the statistical analyses.

## Introduction

Mechanical thrombectomy (MT) is an effective treatment for acute ischemic stroke patients (AIS due to large vessel occlusion (LVO). Rapid and successful reperfusion, defined as thrombolysis in cerebral infarction (TICI) 2b-3, increases the likelihood of a favorable outcome (1, 2). Nevertheless, MT does not result in successful reperfusion in up to 29% of patients (1), and biomarkers that identify patients at risk of failed reperfusion failure are needed.

Clot composition, length, and shape may impact MT success, and imaging predictors of clot response to MT may lead to tailored MT techniques, such as stent-retriever or contact-aspiration, to maximize the likelihood of successful treatment (3, 4). Magnetic resonance imaging (MRI) often demonstrates the thrombus on T2^*^ gradient-echo sequence (GRE) as a region of intravascular hypointense signal abnormality, which is termed the susceptibility vessel sign (SVS). SVS has been used as a measure of clot length to predict response to intravenous thrombolysis (5), to detect small distal occlusions (6), to assess multiplicity of intracranial thrombus fragments (7), and even predict clot composition or stroke etiology (8–13). However, whether SVS depiction of clot shape and extension into vessel branches impacts the likelihood of successful reperfusion has not been investigated. Thrombus that involves a bifurcation or accentuated angle may be more prone to fragmentation and may be more difficult to remove (14).

We hypothesized that SVS may be used to visualize the extent of the clot within the middle cerebral artery branches at the point of vessel occlusion and to determine whether the clot is located in a straight branch (S-SVS) or in an angulated/traversing a bifurcation segment (A-SVS). We determined SVS clot shape, branch occlusion patterns, and the impact of these factors on successful reperfusion and favorable clinical outcomes after thrombectomy for proximal middle cerebral artery occlusions.

## Methods

The study protocol was approved by the institutional review board (IRB) and complied with the Health Insurance Portability and Accountability Act (HIPAA). Patient informed consent was waived by our review board for this single center retrospective analysis of anonymized data acquired prospectively. Adherence to the STROBE criteria (15) was enforced.

### Population and Clinical Data

We performed a retrospective cohort study of consecutive patients who underwent MT treatment for acute ischemic stroke at our comprehensive stroke center between May 2015 and March 2018. Patient inclusion criteria were: (1) pre-MT brain magnetic resonance imaging (MRI) that included an axial T2^*^ sequence [gradient-echo (GRE)], diffusion-weighted imaging (DWI) and perfusion weighted-imaging (PWI) that was free of motion degradation or significant artifact, and (2) middle cerebral artery occlusion (M1 or both M1 and M2). Basilar and internal carotid occlusions were excluded to obtain homogeneous groups and avoid the impact of posterior circulation strokes on the overall outcome.

Clinical and stroke treatment data were determined from a prospectively maintained stroke database and from the electronic medical records. Stroke severity was assessed by the National Institute of Health Stroke Scale (NIHSS) at the time of MT triage. All thrombectomies were performed according to the standard departmental protocols under general anesthesia, using combined stent-retriever (diameter of 6 mm) and contact aspiration with a 6F intermediate catheter, balloon-guided catheters were not used at that time, there were five different attendings.

### Imaging Data and Analysis

All imaging was performed on either a 1.5T GE Signa or 3.0T GE MR750 MRI scanner using standard departmental protocols and approach, using an 8 channel GE HR brain coil (GE Healthcare, Milwaukee, Wisconsin). T2^*^ gradient-echo axial sequences were performed as: TR 650.0 ms, TE 15.0 ms, slice was 5 mm, slice gap of 0.0 mm, FOV of 24.0 × 24.0 cm.

**Figure 1:**
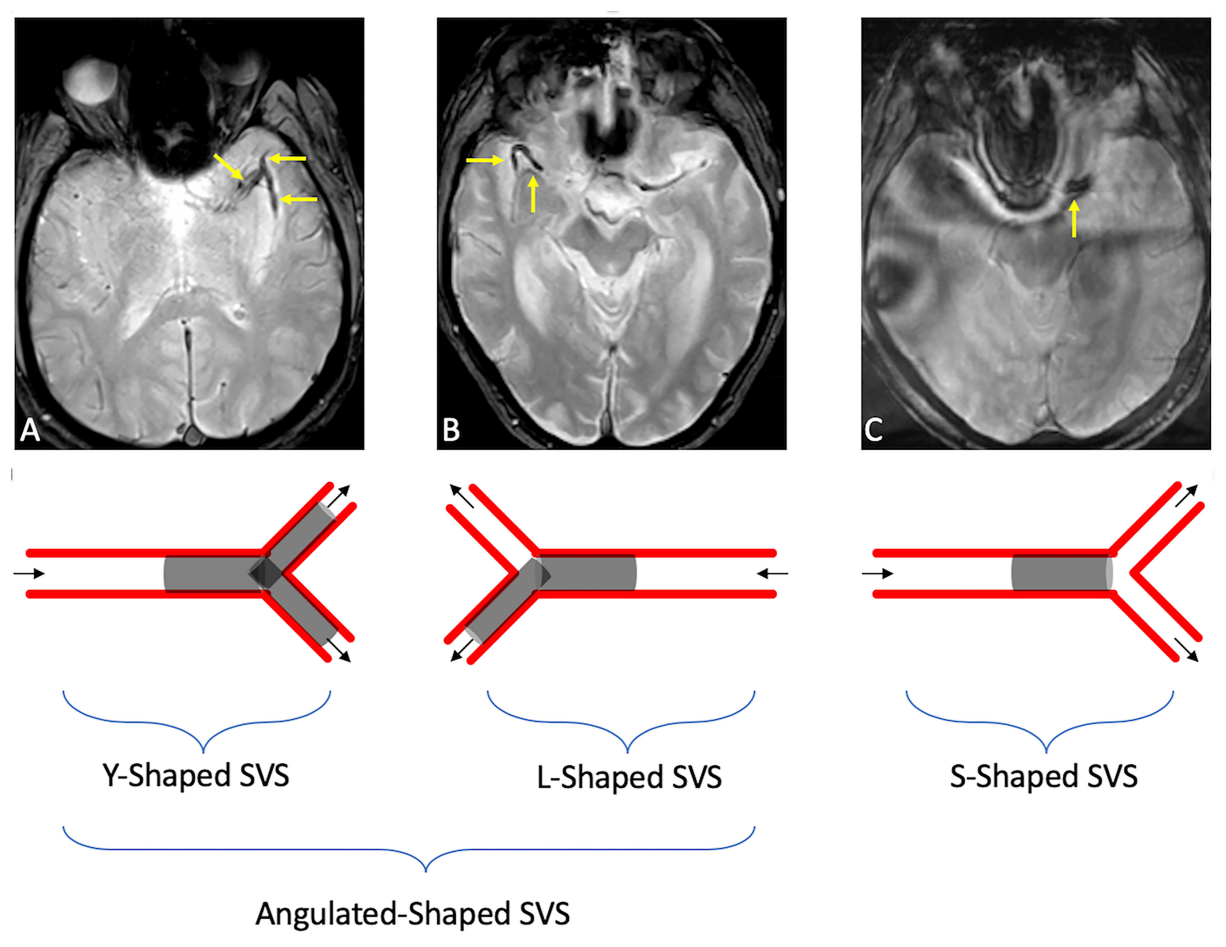
Illustrations of SVS shape in an angulated (A-shaped) branch [either in an MCA bifurcation [a], or an angulated M1–M2 [b]] or in a straight [S-shaped [c]] branch. **(A)** Susceptibility vessel sign in a M1 segment seen as a linear hypo-intensity branch, dividing into two clots within the M2 middle cerebral artery branches. **(B)** Susceptibility vessel sign in a M1 segment seen as a horizontal hypo-intensity in the M1 branch, continuing with a 90° angle in the M1/M2 branch. **(C)** Susceptibility vessel sign in a M1 segment seen as a linear/straight hypo-intensity in the M1 branch (I).

SVS was assessed on GRE sequences and was defined as “presence of a hypointensity within the proximal middle cerebral artery, in which the diameter of the hypointense signal within the vessel exceeded the contralateral vessel diameter” (16). SVS length was measured in millimeters. A-SVS was defined as SVS that involved an angulated M1–M2 segment or in a MCA bifurcation. Clots entirely within a straight M1-segment and without any significant extension into an angulated M2 branch were defined as S-SVS (Figure 1). Patients without SVS were excluded as we couldn't evaluate the clot shape.

MR perfusion-weighted imaging (PWI) data were processed by an automated program (RAPID, iSchemaView, Menlo Park, CA). The ischemic core was defined as the volume of tissue with an apparent diffusion coefficient <620 s/mm^2^. The penumbra was defined as the volume of tissue with a Time-to-maximum (TMax) delay of >6 s. Mismatch volume (17) was assessed as the difference between the ischemic core and the penumbra, and the mismatch ratio (18) was calculated as the ratio between the TMax >6 s lesion volume and the core volume. The hypoperfusion intensity ratio (HIR) was used as a measure of tissue collaterals and was calculated as the volumetric ratio of tissue with a TMax >10 s divided by TMax >6 s (18, 19).

All images were anonymized and blindly analyzed by two neurointerventionalists (A.G. and E.S.S. with 5 and 6 years of experience, respectively). Thrombus length was evaluated from measurements between the proximal and distal extent of the SVS on T2^*^ MR sequences, when the thrombus extended into different branches of the middle cerebral artery, the maximal thrombus length as it extended into one branch vessel was calculated rather than summation of length within all of the involved branches.

Interpretation disagreements were resolved by consensus reading, which was supervised by a third neurointerventionalist (J.J.H. with 10 years of experience).

### Outcomes Measures

Primary outcome was the difference in rate of successful reperfusion (defined as TICI ≥2b) after MT.

Secondary outcomes were MT complication rates, MT reperfusion time (minutes), and clinical outcomes. Early clinical outcomes were assessed using NIHSS on day 1 post-MT (POD1), NIHSS shift between baseline and POD1, and discharge NIHSS. Long term clinical outcomes were assessed using the modified Rankin Scale (mRS) 90-days after MT; excellent clinical outcome was defined as mRS 0–1, good clinical outcome was defined as mRS 0–2, and poor outcome was defined as mRS 3–6.

Recorded MT complications included emboli to a new vascular territory, arterial perforation, or symptomatic hemorrhage, which was defined as a parenchymal hematoma (PH1 or PH2) with associated worsening from the baseline NIHSS of at least four points.

### Statistical Analysis

Nominal variables were first summarized using frequency descriptive analysis then compared using Fisher's exact test. Continuous variables were summarized using median, quartiles and interquartile range, then tested on univariate analysis using the Mann-Whitney test. Normality of the variables was tested by the Shapiro-Wilk test. Statistical significance was set at the *p* = 0.05 level.

Logistic regression models were designed to assess the association of SVS clot-shape with successful reperfusion (TICI ≥2b). To adjust for baseline and MT potential confounders, a multivariate binary logistic regression analysis was conducted.

Factors with a significant association (*P* < 0.10) in the univariate analysis (clot length) were included in the multivariable model and factors associated with patient's outcome in the literature were forced into. Results were expressed as odds ratios (ORs) and 95% confidence intervals (CIs) using S-SVS as reference group.

Other logistic regression models were subsequently designed to assess the association of SVS clot-shape with favorable clinical outcome (mRS 0–2), excellent clinical outcome (mRS 0–1), and mortality (mRS 6).

Initial agreement between the two interventional neuroradiologists was measured using Kappa of Cohen, then disagreements were resolved by consensus reading.

All statistical analyses were performed with XLSTAT (Addinsoft, New York City, NY).

### Data Availability Statement

The data that support the findings of this study are available from the corresponding author upon reasonable request.

## Results

**Figure 2:**
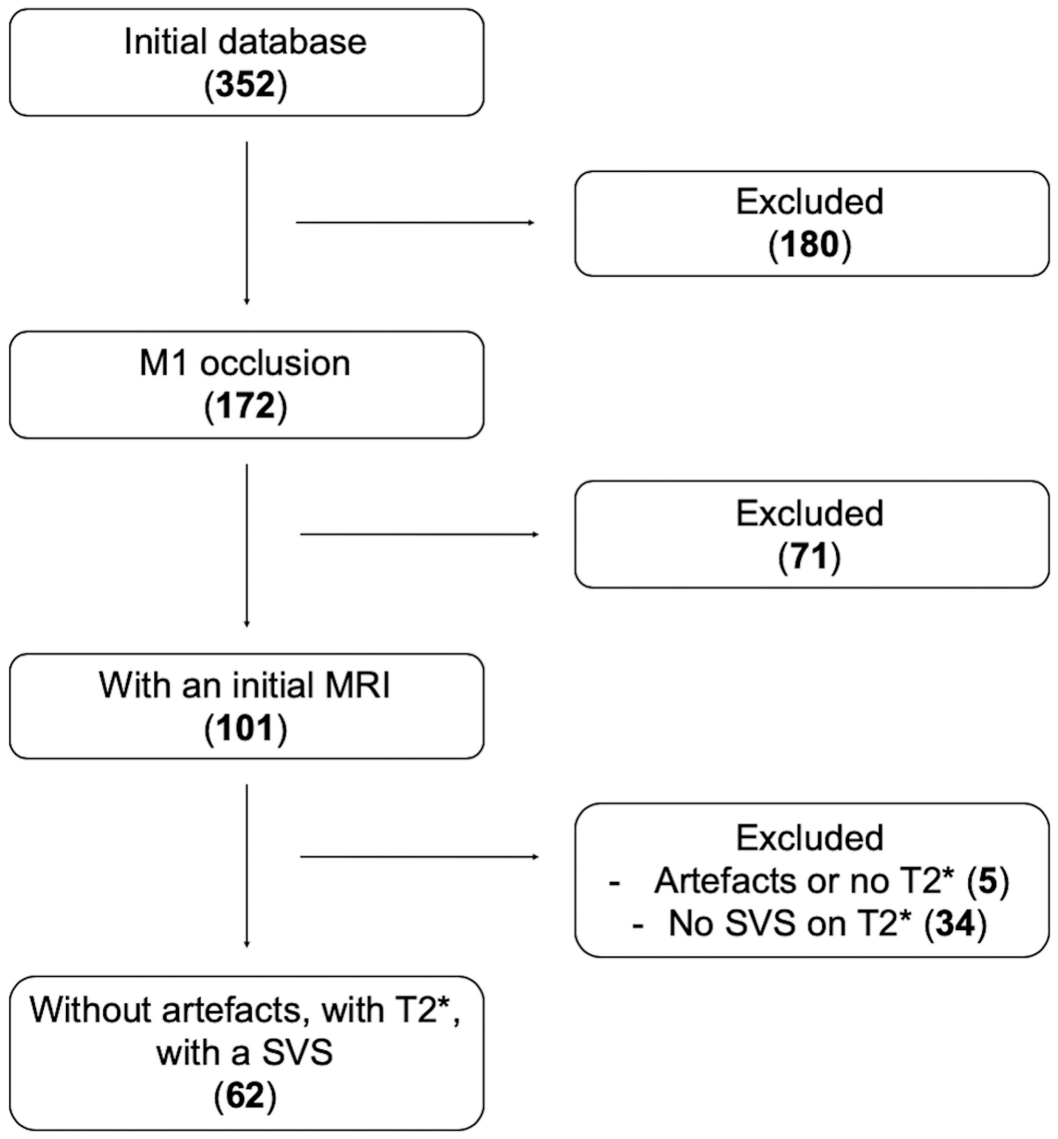
Flow-chart. Number of patients screened then included according to our inclusion criteria: Initial database 352 patients; among them 172 had a proximal MCA (M1) occlusion; 101 of them were screened by MRI, and 71 were screened by CT scan and excluded; 62 patients had A SVS on their GRE imaging.

Sixty-two patients met inclusion criteria (Figure 2). Patients were dichotomized into S-SVS (27 patients; 44%) and A-SVS (35 patients; 56%) groups; reader agreement of SVS classification was substantial (Cohen's Kappa 0.711) (20).

There were no differences between S-SVS and A-SVS with respect to patient age [72 (IQR, 63–79) vs. 67 (IQR, 55–76), *p* = 0.960], female sex (52 vs. 54%, *p* = 1.000), baseline NIHSS [14 (IQR, 10–20) vs. 17 (IQR, 13–22), *p* = 0.12], or frequency of left sided occlusions (71 vs. 54%, *p* = 0.19), respectively (Table 1). A-SVS clots were significantly longer compared to S-SVS clots [19 mm (IQR, 15–24) vs. 8 mm (IQR, 10–15), *p* = 0.0002] (Table 2). There were no other significant differences between the two groups with regard to neuroimaging variables, which included core infarction volume, penumbra volume, and HIR collateral robustness (Table 2). Likewise, MT procedural outcomes were similar between S-SVS and A-SVS groups (Table 3). There were no differences in time from groin puncture to reperfusion [36 min (IQR, 25–62) vs. 44 min (IQR, 23–77), *p* = 0.70] or MT complication rates (4 vs. 11%, *p* = 0.27).

**Table 1:** Baseline characteristics for patients with an A-shaped SVS compared to those with an S-shaped SVS (S-SVS).

	**All**	**S-SVS**	**A-SVS**	***p*-value**
Number of patients	62/62 (100%)	27/62 (44%)	35/62 (56%)	
Age, years (median, IQR)	70 (57–77)	72 (63–79)	67 (55–76)	0.960
Female (%)	33 (53%)	14 (52%)	19 (54%)	1.000
**Medical history**				
Hypertension (%)	44 (71%)	18 (67%)	26 (74%)	0.51
Diabetes (%)	16 (26%)	8 (30%)	8 (23%)	0.41
Hyperlipidemia	30 (48%)	11 (41%)	19 (54%)	0.33
Atrial fibrillation (%)	31 (50%)	15 (56%)	16 (46%)	0.34
Coronary artery disease (%)	13 (21%)	6 (22%)	7 (20%)	0.49
Prior stroke (%)	8 (13%)	3 (11%)	5 (14%)	0.71
Platelets	195 (163–228)	207 (171–240)	179 (161–212)	0.15
Smoking				0.16
Never (%)	35 (57%)	17 (63%)	18 (51%)	
Prior (%)	19 (31%)	9 (33%)	10 (29%)	
Current (%)	8 (13%)	1 (4%)	7 (20%)	
**Stroke details**				
Baseline NIHSS (median, IQR)	16 (12–21)	14 (10–20)	17 (13–22)	0.12
Drugs and medications				
Antiplatelet or anticoagulant	28 (45%)	15 (56%)	13 (36%)	0.15
Intravenous tPA (%)	40 (65%)	18 (67%)	22 (63%)	0.48

**Table 2:** Imaging characteristics for patients with an A-shaped SVS compared to those with an S-shaped SVS.

	**All**	**S-SVS**	**A-SVS**	***p*-value**
Number of patients (%)	62/62 (100%)	27/62 (44%)	35/62 (56%)	
**MRI characteristics**				
Core volume, ml (median, IQR)	15 (6–34)	11 (5–38)	15 (7–31)	0.66
Penumbra TMax >6 s volume, ml (median, IQR)	101 (66–127)	97 (66–117)	104 (68–132)	0.59
Penumbra TMax >10 s volume, ml (median, IQR)	32 (18–54)	28 (15–44)	32 (22–70)	0.38
Mismatch Volume, ml (median, IQR)	71 (51–102)	71 (55–93)	75 (51–107)	0.73
Mismatch ratio (median, IQR)	6 (3–16)	7 (3–17)	5 (3–15)	0.89
HIR (median, IQR)	0.37 (0.23–0.48)	0.32 (0.21–0.42)	0.39 (0.26–0.52)	0.26
Clot length, mm, (median, IQR)	15 (10–20)	8 (10–15)	19 (15–24)	0.0002
**Vessel occlusion side**				
Left (%)	38 (61%)	19 (71%)	19 (54%)	0.19

**Table 3:** Outcomes for patients with an A-shaped SVS compared to those with an S-shaped SVS.

	**All**	**S-SVS**	**A-SVS**	***p*-value**
Number of patients (%)	62/62 (100%)	27/62 (44%)	35/62 (56%)	
**MT**				
TICI 2b/2c/3 (%)	55 (89%)	26 (96%)	29 (83%)	0.09
TICI 2c/3 (%)	31 (50%)	16 (59%)	15 (43%)	0.20
TICI 0 (%)	0 (0%)	0 (0%)	0 (0%)	1
TICI 1 (%)	1 (2%)	0 (0%)	1 (3%)	0.38
TICI 2a (%)	6 (10%)	1 (4%)	5 (14%)	0.16
TICI 2b (%)	24 (39%)	10 (37%)	14 (40%)	0.81
TICI 2c (%)	13 (21%)	7 (26%)	6 (17%)	0.40
TICI 3 (%)	18 (29%)	9 (33%)	9 (26%)	0.51
Complications (%)	5 (8%)	1 (4%)	4 (11%)	0.27
Groin/reperfusion time (min)	40 (24–71)	36 (25–62)	44 (23–77)	0.70
Onset/reperfusion time (min)	419 (306–519)	418 (249–545)	420 (312–484)	0.71
**Early clinical outcome**				
24 h NIHSS (median, IQR)	10 (5–16)	5 (2–15)	11 (7–18)	0.03
NIHSS SHIFT (median, IQR)	−7 (−11 to −1)	−6 (−10 to −2)	−7 (−11 to 0)	0.67
Discharge NIHSS (median, IQR)	5 (2–13)	3 (2–9)	8 (3–18)	0.004
**Long-term clinical outcome (55/62)**				
3 months good mRS (0–1–2) (%)	28/55 (51%)	14/25 (56%)	14/30 (47%)	0.49
3 months excellent mRS (0–1) (%)	18/55 (33%)	11/25 (44%)	7/30 (23%)	0.10
3 months mortality (%)	13/55 (23%)	5/25 (20%)	8/30 (26%)	0.56

All other univariate analysis are described in Table 3.

In the multivariable binary logistic regression model (AUC = 0.893), A-SVS was an independent negative predictor of successful reperfusion [OR 0.04 (95% CI, 0.002–0.58), *p* = 0.02, Table 4].

**Table 4:** Multivariate analysis.

	**S-SVS (*n* = 27)**	**A-SVS (*n* = 35)**	**Unadjusted OR (95% CI)**	**Unadjusted *p*-value**	**Adjusted OR (95% CI)**	**Adjusted *p*-value**
**Technical details**						
Successful reperfusion	96% (26/27)	83% (29/35)	0.23 (0.03–2.10)	*p* = 0.194	0.04 (0.002–0.58)	*p* = 0.02
(TICI ≥2b)^*^						
**Clinical outcomes**						
Good clinical outcome	56% (14/25)	47% (14/30)	0.69 (0.24–1.99)	*p* = 0.49	0.72 (0.21–2.46)	*p* = 0.59
(mRS 0–2)^**^						
Excellent clinical outcome	44% (11/25)	23% (7/30)	0.39 (0.12–1.23)	*p* = 0.11	0.35 (0.09–1.46)	*p* = 0.15
(mRS 0–1)^**^						
Mortality	20% (5/25)	26% (8/30)	1.46 (0.41–5.18)	*p* = 0.56	3.22 (0.67–15.49)	*p* = 0.14
(mRS 6)^**^						

* *Effect of clot shape, adjusted for baseline NIHSS, clot length, with S-SVS as reference group*.

** *Effect of clot shape, adjusted for baseline NIHSS, collaterals [HIR], clot length, baseline infarct volume, with S-SVS as reference group*.

There was no impact of A-SVS clot shape in the multivariable binary logistic regression models on good clinical outcome [OR 0.72 (95% CI, 0.21–2.46), *p* = 0.59], excellent clinical outcome [OR 0.35 (95% CI, 0.09–1.46), *p* = 0.15], or mortality [OR 3.22 (95% CI, 0.67–15.49), *p* = 0.14], respectively (Table 4).

## Discussion

In this study, we found that thrombus morphology measured by SVS-shape influences the likelihood of successful reperfusion after MT. However, SVS morphology did not affect the likelihood of achieving a favorable clinical outcome. Our findings that SVS thrombus morphology is a biomarker of reperfusion have important implications for MT.

Prior studies have used SVS identified on gradient-echo imaging (T2^*^) (21) to detect (5), localize (22), and measure clot length (5, 22) without contrast administration. Whether SVS is a predictor of reperfusion after MT remains controversial (9, 23). Our findings support the hypothesis that SVS is a biomarker of reperfusion when thrombus morphology is considered. S-SVS are linear clots that do not extend into arterial branch vessels, and clots with this morphology were likely to undergo complete reperfusion compared to angulated clots and clots that extend into branch vessels (A-SVS). Whether a prospective change to MT technique results in higher rates of reperfusion of A-SVS clots requires further study. While clot morphology will not impact the decision to perform a thrombectomy procedure, we hypothesize that it could impact technical strategy, and the routine use of balloon guide sheaths, longer stentretrievers, or even double stentretriever techniques (placement of two devices into two branch points involved with a A-SVS clot) may increase the likelihood of complete reperfusion of A-SVS clots (24, 25).

Patients selection for MT depends on a fast identification of a LVO and on the evaluation of early ischemic changes, and computed-tomography (CT) and magnetic-resonance-imaging (MRI) are recommended for patient evaluation (26). However, MRI is superior to CT for detection of acute ischemia (27), is associated with better outcomes after thrombectomy treatment (28), whereas CT has been associated with an increased risk of futile reperfusion (29). While MRI duration is often reported to be longer in patient's screening for MT (30), MRI did not delay MT (30) nor impact patient's functional outcome in recent studies (30). Use of MRI in AIS screening may then depend on local protocols and optimal institutional workflows.

In contrast to few prior studies that evaluated the importance of clot length on reperfusion (22, 31) our study focused on clot morphology only. In contrast to intravenous thrombolysis stroke treatment, the impact of clot length on successful reperfusion after MT remains controversial (32, 33) and requires further study.

Successful reperfusion has been correlated to clinical outcome in multiple studies (34, 35) and, therefore, one would expect S-SVS to be correlated with a greater likelihood of a favorable clinical outcome. In our study, S-SVS patients had a lower NIHSS the day after thrombectomy and at discharge, but this early recovery did not translate to better outcomes at 90 days. We hypothesize that our study is under powered to detect an outcome difference between S-SVS and A-SVS patients.

### Limitations

Our study is limited by its retrospective observational and single center design, which may introduce bias. The relatively small sample size and missing clinical outcomes in 7/62 patients may also introduce bias in our secondary outcome analysis, our findings need to be confirmed in a larger prospective study. The use of GRE MRI to identify SVS and SVS morphology rather than volumetric susceptibility or CT techniques as well as the exclusion of patients without SVS may limit the generalizability of our findings.

## Conclusion

SVS clot morphology appears to be an independent predictor of successful reperfusion after MT in our cohort.

## Data Availability Statement

The raw data supporting the conclusions of this article will be made available by the authors, without undue reservation.

## Ethics Statement

The studies involving human participants were reviewed and approved by Stanford Medical Center Committee. Written informed consent for participation was not required for this study in accordance with the national legislation and the institutional requirements.

## Author Contributions

AG, RF, ES, ML, GA, BM, DM, GK, MM, ML, MW, and JH participated to study design, data collection, data analysis, and writing. All authors contributed to the article and approved the submitted version.

## Conflict of Interest

GA reports equity and consulting for iSchemaView and consulting from Medtronic. MM reports Ownership Interest in ThrombX Medical. JH reports Consultant or Advisory Board for Medtronic, Inc., MicroVention, Inc., and iSchemaView. The remaining authors declare that the research was conducted in the absence of any commercial or financial relationships that could be construed as a potential conflict of interest.

## Abbreviations

AIS: Acute Ischemic Stroke
A-SVS/S-SVS: patients with an angulated or bifurcation-shaped (A-shaped) Susceptibility Vessel Sign/patients with a straight (S-shaped) Susceptibility Vessel Sign
M1: Proximal Middle Cerebral Artery
mRS: modified Rankin Scale
MT: Mechanical Thrombectomy
NIHSS: National Institute of Health Stroke Scale
POD1: Post-Operative Day 1 post-MT
SVS: Susceptibility Vessel Sign
TICI: Thrombolysis In Cerebral Infarction
TMax: Time to Maximum (sec)
CT: Computed Tomography
HIR: Hypoperfusion Intensity Ratio
ICA: Internal Carotid Artery
ICA T: Internal Carotid Artery Termination
IQR: Inter-Quartile Range
LVO: Large Vessel Occlusion
MCA-M1: Proximal Middle Cerebral Artery
MRI: Magnetic Resonance Imaging
tPA: thrombolysis Plasminogen Activator.
